# The Role of *Phragmites australis* in Mediating Inland Salt Marsh Migration in a Mid-Atlantic Estuary

**DOI:** 10.1371/journal.pone.0065091

**Published:** 2013-05-21

**Authors:** Joseph A. M. Smith

**Affiliations:** Nature Conservancy, Delmont, New Jersey, United States of America; Bangor University, United Kingdom

## Abstract

Many sea level rise adaptation plans emphasize the protection of adjacent uplands to allow for inland salt marsh migration, but little empirical information exists on this process. Using aerial photos from 1930 and 2006 of Delaware Estuary coastal habitats in New Jersey, I documented the rate of coastal forest retreat and the rate of inland salt marsh migration across 101.1 km of undeveloped salt marsh and forest ecotone. Over this time, the amount of forest edge at this ecotone nearly doubled. In addition, the average amount of forest retreat was 141.2 m while the amount of salt marsh inland migration was 41.9 m. Variation in forest retreat within the study area was influenced by variation in slope. The lag between the amount of forest retreat and salt marsh migration is accounted for by the presence of *Phragmites australis* which occupies the forest and salt marsh ecotone. *Phragmites* expands from this edge into forest dieback areas, and the ability of salt marsh to move inland and displace *Phragmites* is likely influenced by salinity at both an estuary-wide scale and at the scale of local subwatersheds. Inland movement of salt marsh is lowest at lower salinity areas further away from the mouth of the estuary and closer to local heads of tide. These results allow for better prediction of salt marsh migration in estuarine landscapes and provide guidance for adaptation planners seeking to prioritize those places with the highest likelihood of inland salt marsh migration in the near-term.

## Introduction

Coastal habitats are dynamic by nature, but sea level rise resulting from climate change has the potential to dramatically alter the pace of these dynamics [1]. There is increasing recognition that planning for adaptation to sea-level rise will require that upland areas adjacent to salt marshes be available for these marshes to transgress inland [2]–[4]. This strategy is intended to help offset losses of salt marsh due to coastal erosion, and when sea level rise outpaces the accretion capacity of these marshes [5]–[8]. The process of habitat change at this upland interface is well-understood over geologic time-scales [9]–[11], but there are few studies that document this process at a contemporary time-scale [12], [13]. Such information is needed to gain a realistic understanding of the contribution these upland areas will make toward offsetting salt marsh losses due to sea level rise.

On the east coast of North America, there has been a steady pace of sea level rise that gradually overtakes forested uplands and wetlands [14]. Over time, these areas become part of the salt marsh as salinity and moisture increase at upland edges [3]. Reports of losses to the land base from eyewitness accounts date at least as far back as the mid- 19th century [15]. These recent losses of terrestrial habitats to sea level rise present an opportunity to examine how the change from forest to salt marsh proceeds over time so that future changes can be better anticipated.

Coastal wetland and upland habitats comprise a mosaic that is formed by variation in salinity and inundation regimes [16], [17]. These habitats, moving from sea to land, transition from open water and tidal flats to salt-tolerant emergent marshes, and eventually to forest [16]. Additionally, in many locations in the eastern United States, *Phragmites australis* occupies a band at the upper border of salt marshes at the forest ecotone [18]–[21]. This phenomenon is most common in tidal marshes with salinity levels (>18) because *Phragmites* is limited by salinity, which under most circumstances [22]–[24] prevents it from further expanding into these marshes [25].

The introduction of a non-native genotype of *Phragmites* in the early 19th century transformed this species to a dominant species in many wetland habitats, particularly in disturbed areas [20], [24], [26]. One example of such a disturbance is when forest fringing the coast is killed by encroaching sea levels [12], [27]. This process creates an opportunity for colonization of these vacant areas by *Phragmites* which is better adapted to the resulting brackish conditions [24].

In this study, I use aerial photos to examine how habitat change at the forest-salt marsh interface has proceeded in recent decades (between 1930 and 2006) along the coast of the Delaware Estuary. I track the process of habitat transition from forest to saline emergent wetland habitats by measuring the amount of forest loss as well as the amount of inland salt marsh migration in corresponding areas. I then examine the role of salinity and local topography in influencing patterns of change and determine the role that *Phragmites* plays as a mediator of change.

## Methods

### Study Site

This work focused on the Delaware Bay coast of New Jersey in Cumberland and Cape May counties (39°9′, 74°54′). The low level of coastal development in this region makes it an ideal place to study the dynamics of salt marsh-upland ecotones over time. The region is experiencing an average rate of relative sea level rise of approximately 3 mm per year [28] due to both post-glacial subsidence and global sea level rise [14]. The sea level rise experienced by the region has resulted in a dynamic land-sea interface where upland habitats yield to saline wetlands over time [15], [27]. Mean tidal range in the study area is 1.7 m and ranges from 1.4 to 1.8 m (tidesandcurrents.noaa.gov).

I selected all segments of the upland-salt marsh interface along the New Jersey coast of the Delaware Bay that have remained forested or have reverted to forest from agriculture since 1930. I also inspected bare-earth LIDAR digital elevation models (available from NOAA Digital Coast, http://www.csc.noaa.gov/digitalcoast/) to identify and exclude from analysis any upland-salt marsh ecotones with raised features that might interfere with change processes such as former and current dykes, and roadbeds. Total linear amount of forest edge included in this study is 101.1 Kilometers.

Within this area, tidal salt marsh habitats are dominated by *Spartina alterniflora*, with *Spartina patens* and *Distichlis spicata* occurring in mixed patches throughout the marsh. Higher areas of marsh near the forested upland edge transition directly into monotypic stands of *Phragmites australis*.

The fringing band of *Phragmites* at the transition zone between salt marsh and forest in this region has been present since at least 1910 [29]. This band ranges in width from less than 10 m up to 500 m wide across the study sites. *Phragmites* is limited by salinity in these locations and my observations indicate that it does not encroach on the salt marsh above salinity levels that are greater than 18 at our study sites [25]. Average estuary salinities across the study area ranges from 17 to 30 [30].

Forests across these study sites are primarily hardwood forest (characteristic tree species *Acer rubrum*, *Liquidambar styraciflua*, *Nyssa sylvatica*, *Quercus phellos, Quercus alba, Ilex opaca, Vaccinium corybosum*) and Atlantic white cedar (*Chamaecyparis thyoides*) peat swamp.

### Habitat change mapping

To examine the change in forest extent between 1930 and 2006, I hand-digitized the forest edge at both of these intervals using aerial photography. For the 1930 interval, I used black and white aerial photography which was digitized (2 m resolution) and rectified by the State of New Jersey Office of Information Technology, Office of Geographic Information Systems (https://njgin.state.nj.us). For the 2006 interval, I used USDA National Agriculture Imagery Program 1 m resolution true-color aerial imagery (http://datagateway.nrcs.usda.gov/). I estimated the accuracy of 1930 imagery against 2006 imagery by selecting control points at road intersections that were present during both time intervals. Based on 30 point pairs distributed throughout the study region, the root mean squared error (RMSE) is 11.3 m.

I also used the 2006 imagery to map the fringing band of *Phragmites* that exists between the forest and salt marsh. This growing season imagery shows a distinct color contrast between *Phragmites* and salt marsh [31], [32]. When mapping *Phragmites*, I frequently referred to oblique aerial photos available via Bing maps (bingmaps.com; “bird's eye view”) which revealed contrasts in vegetation height [33] that helped confirm mapping decisions. In August of 2011, I visited 17 of the 41 study catchments to ground-truth mapping work, particularly to confirm the accuracy phragmites-saltmarsh edge mapping and to search for evidence of tree stumps in marsh areas that were forested during the 1930 time frame.

To calculate change in habitat extents, I used the Digital Shoreline Analysis System (DSAS) [34] extension for Arcview 9.3 (ESRI Inc., Redlands, CA). This tool computes change statistics for habitat boundaries measured at discrete occasions. In this case, these boundaries were the forest-emergent wetland boundary in 1930 and 2006, as well as the salt marsh-*Phragmites* boundary in 2006.

DSAS generates a series of evenly spaced transects perpendicular to a user-supplied baseline. I hand-digitized a baseline that paralleled the coast-ward edge of the forest in 1930. Once transects were generated (starting at the beginning of the baseline and spaced 25 m apart), I excluded any transects that were not perpendicular to the boundary orientation. This occurred occasionally when habitat boundaries curved sharply. DSAS marks the intersections where these transects (n = 1523) crossed mapped habitat boundaries. From these intersections, I measured the amount that the forest edge receded between 1930 and 2006. I used the “end point rate” measure which calculates the distance between intersections.

**Figure 1:**
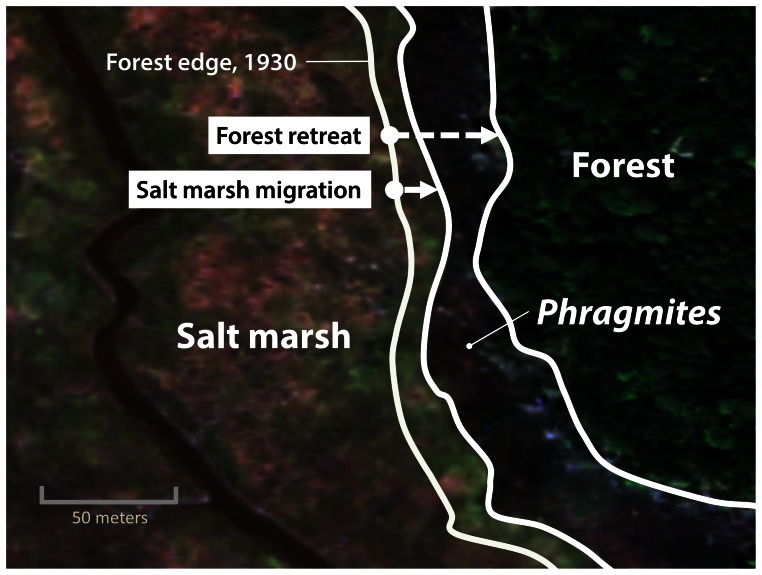
Illustration of the approach for measuring forest retreat and salt marsh migration. Forest retreat is the distance between the 2006 forest edge and 1930 forest edge. Salt marsh migration is the proportion of the forest retreat zone that was occupied by salt marsh in 2006. Image from 2006 USDA National Agriculture Imagery Program.

To measure salt marsh migration inland, I focused on the zone of forest retreat from 1930–2006 and used the transects to calculate (1) the width of the Phragmites band at the forest-salt marsh ecotone in 2006; and (2) the width of the salt marsh within the zone of forest retreat (Figure 1). The measure of salt marsh migration is expressed as the percent of the transect that was formerly forest in 1930 that was salt marsh in 2006.

### Predictors of change

I examined several covariates in relation to these measures of change. These were slope, and two proxies for salinity: distance up the estuary along the coast from the mouth of the Delaware Bay at Cape May, NJ and distance from head of tide along coastal sub-watersheds.

I created a slope metric from digital elevation models derived from LiDAR data (available from NOAA Digital Coast, http://www.csc.noaa.gov/digitalcoast/) and calculated average slope between the 1930 forest edge and the 2007 forest edge along the transects created using DSAS (described above).

As a proxy for the estuary-scale salinity gradient, I used distance along the coast from the mouth of the Delaware Bay at Cape May, NJ. I chose relative distance up the bay as a proxy because salinity in the estuary varies annually, seasonally, and over longer time scales, making it challenging to arrive at an average representation of salinity for the 86-year period we examined. This bay-scale distance measure nonetheless correlated strongly with 5×5 km resolution salinity maps [35] for the bay that was derived from National Oceanographic Data Center data averaged across depths, seasons, and years (1950–2000; R^2^ = 0.87, df = 65, P<0.0001, n = 66 5 km quadrants).

To capture a second, local gradient in salinity that occurs in individual subwatersheds that fringe the bay, I considered distance from head of tide as a covariate. These watersheds decrease in salinity as they reach upstream areas of emergent marshes. I used a point layer available from the NJ Department of Environmental Protection (1986, http://www.nj.gov/dep/gis) to represent head of tide. All head of tide points for subwatersheds and their tributaries are mapped in this layer for tidal streams of NJ. For each observation, the euclidean distance to the nearest head of tide location was recorded. These head of tide markers are a relative measure because, due to local sea level rise, they were likely to be positioned further downstream at the start of the analysis period in 1930, and are now likely to be upstream of their marked location in 1986. I confirmed the presence of salinity gradients in a subset of coastal streams with field measurements using a temperature and conductivity meter (YSI model 33 S-C-T) during August 2011. I sampled 7 tidal streams by launching a canoe at a downstream location with a salinity equal to the adjacent Delaware Bay. Sampling was timed to coincide with the end of the rising tide so that measurements occurred during slack tide. I sampled salinity mid-channel every 250 meters and ascended streams until salinity measurements were zero. The local-scale distance metric correlated strongly with field salinity measurements. In a regression model that also included the estuary-scale salinity proxy as a covariate (P<0.001), salinity increased with increasing distance from head of tide (R^2^ = 0.66, df = 66, P<0.001, n = 69).

I summarized all variables, including measurements of habitat change, by National Hydrography Dataset (NHDplus) catchment units. These catchment units delineate the drainage areas for separate watershed stream reaches in the NHDplus dataset and allowed us to group observations into ecologically-relevant landscape units that were less likely to be spatially autocorrelated. The study area comprised 41 catchment units (average size of catchments, 30.5 hectares, range 7 to 200 hectares), with individual units having a mean of 36.3 (±1.3SE) individual transect observations. Based on 2006 landcover maps [36], forest composition in these catchments is dominated by hardwood forest (n = 37), with a smaller proportion dominated by white cedar peat swamp (n = 4). For each catchment, we calculated the average amount of forest dieback and marsh migration, along with averages for slope and salinity-proxy covariates. All subsequent analyses were conducted at the catchment level.

To examine the role that slope and salinity play in determining rates of forest dieback and marsh migration, I first performed diagnostics to test for normality and spatial autocorrelation among catchment units. I performed these diagnostics in program Geoda version 0.9 (geodacenter.asu.edu/software/downloads). None of the independent variables included in the models below were significantly correlated and the two dependent variables in the separate analyses below were also not correlated.

For the analysis of forest dieback, I used the generalized linear model to examine the influence of salinity and slope on the amount of forest dieback. This analysis used average amount of forest dieback by catchment (n = 41) as the response variable. For predictor variables I used catchment-level averages of the slope metric and two salinity metrics described above. An interaction term between the two salinity variables was also included in the model. Diagnostic examination of regression residuals indicated that the gamma distribution was most appropriate. I implemented this model in program R using a log link function. Moran's I test (using a first-order queen contiguity spatial weights matrix in Geoda) indicated that the model outcome was spatially independent (z = −.56, p = 0.58).

For the analysis of salt marsh migration, I used the generalized linear model with a normal distribution and identity link function to examine the influence of salinity and slope on the amount of inland marsh migration. This analysis used the salt marsh migration estimate (proportion of former forest along transects now occupied by salt marsh) averaged by catchment (n = 41) as the response variable. For predictor variables I used catchment-level averages of the slope metric and two salinity metrics described above. An interaction term between the two salinity variables was also included in the model. All variables met parametric assumptions, and a Moran's I test indicated that the model outcome was spatially independent (z = 0.60, p = 0.55)

## Results

I analyzed 101.1 kilometers of forest-salt marsh ecotone along the Delaware Bay as measured in 2006. Total edge of this ecotone increased from 1930 to 2006. In 1930, the corresponding edge length was 51.7 kilometers.

### Rate of forest dieback and marsh migration

Across the study sites, forests receded an average of 141.2±22.8 meters SE (equivalent to 1.8 m/yr, n = 41 catchments) during the 1930 to 2006 time period. The small sample of Atlantic white cedar-dominated catchments (n = 4) experienced a significantly greater (Mann-Whitney U test, U = 13, p = 0.004) amount of forest dieback (mean = 372.3±62.8 meters SE) compared with hardwood forest-dominated catchments (n = 37, mean = 116.2±20.6 meters SE). Over this same time period, inland migration of salt marsh into this formerly forest area averaged 41.9±8.9 meters SE (equivalent to 0.54 m/yr, n = 41 catchments). Salt marsh migration inland did differ significantly between Atlantic white cedar and hardwood-dominated catchments (Mann-Whitney U test, U = 31, p = 0.06). The remainder of the former forest was occupied by *Phragmites*. Overall, 32.3% (±0.03% SE) of the former forest area was occupied by salt marsh in 2006.

### The role of slope and salinity

#### Forest dieback

**Figure 2:**
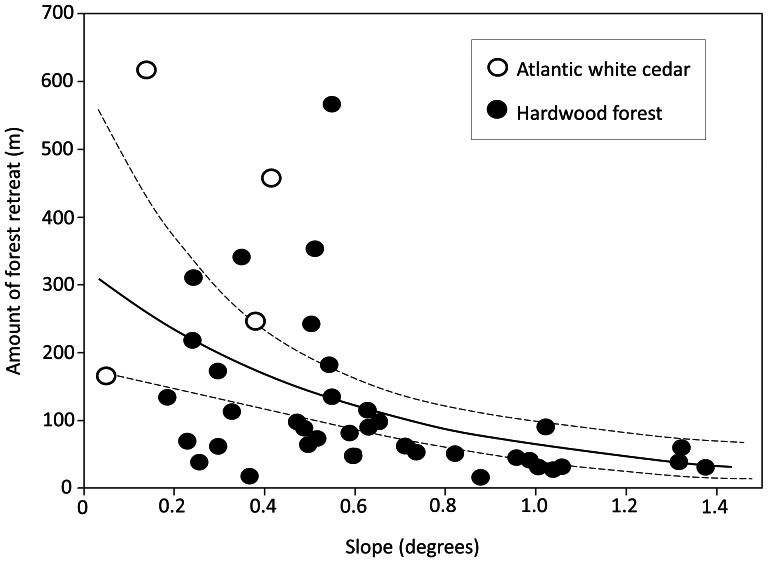
Relationship between slope and the amount of forest retreat between 1930 and 2006. Solid line is the predicted value from a Generalized Linear Model with dotted lines representing 95% confidence intervals. See Table 1 for statistical details.

Slope was a key determinant of variation in forest loss. In a model that included estuary-wide salinity gradient, local salinity gradient, the interaction between these two gradients, and slope (*X^2^* (4, 41) = 30.55, p<0.0001), only slope was significant (p<0.001, Table 1). Forest dieback was lowest at steeper slopes (Figure 2). Slope among samples ranged from 0.05° to 1.38° (Mean = 0.60±0.05° SE). Atlantic white cedar-dominated catchments had significantly less steep slopes (Mean = 0.25±0.16° SE, n = 4) than hardwood forest-dominated catchments (Mean = 0.64°±0.05° SE, n = 37.

**Table 1:** Results of Generalized Linear Model for the effect of slope and salinity on the amount of coastal forest retreat between 1930 and 2006.

Independent variable	Estimate	Std. Error	*t-Value*	*p-Value*
Intercept	7.702779	1.233548	6.244	0.0000
Estuary-wide salinity gradient	−0.01193	0.012655	−0.943	0.3520
Local salinity gradient	−0.44786	0.52385	−0.855	0.3980
Local x estuary-wide salinity gradient	0.001251	0.005926	0.211	0.8340
Slope	−1.72451	0.384389	−4.486	0.0001

#### Salt marsh migration

**Figure 3:**
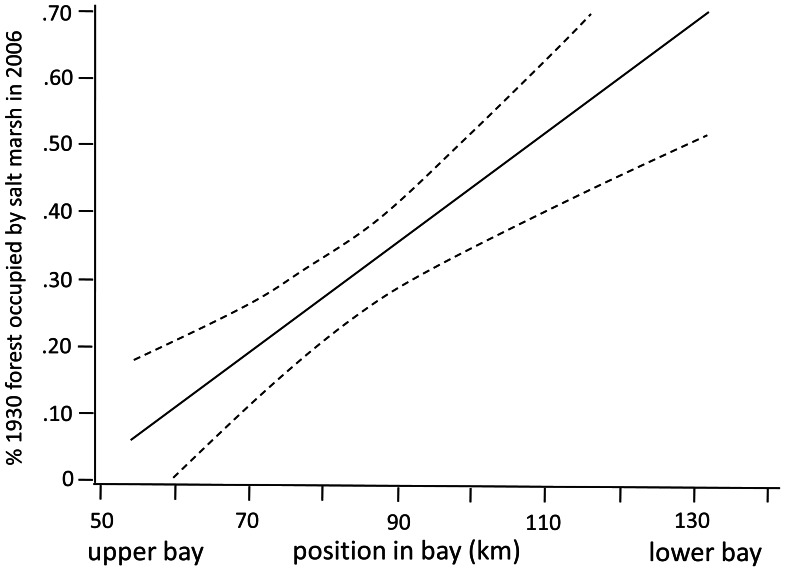
Relationship between location along coast (a proxy for the estuary-wide salinity gradient) and proportion of former forest that became salt marsh between 1930 and 2006. Solid line is the predicted value from a generalized linear model, with dotted lines representing 95% confidence intervals. See Table 2 for statistical details.

**Figure 4:**
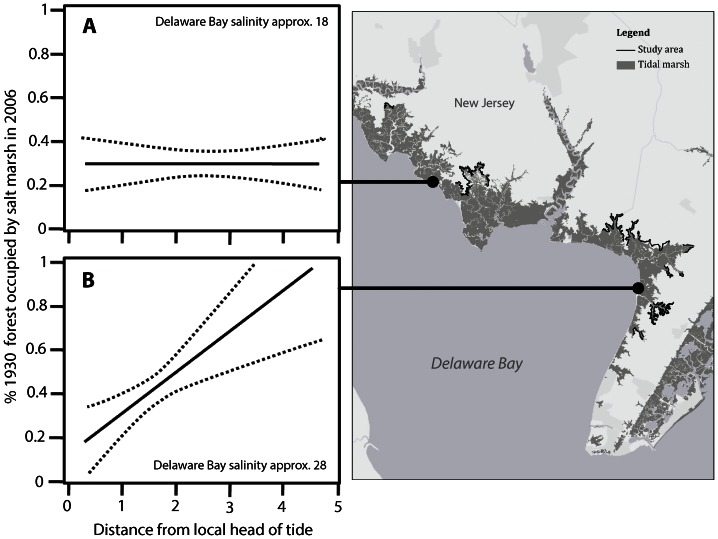
Relationship between local head of tide (a proxy for local-scale salinity gradients along sub-watersheds) and the proportion of former forest that became salt marsh between 1930 and 2006 after accounting for the interaction between the effect of estuary-wide salinity (see Figure 3). The relationship is depicted at two example locations to illustrate the effect of the interaction: (A) further away from the mouth of the bay where bay salinity is approximately 18 and (B) depicts this relationship closer to the mouth of the bay in a higher-salinity setting (approximately 28). See table 2 for statistical details.

The proportion of former forest currently occupied by salt marsh (i.e. salt marsh migration) varied across the landscape. Slope and salinity explained much of this variation. In a model that included estuary-wide salinity gradient, local salinity gradient, the interaction between these two gradients, and slope (*X^2^* (4, 41) = 30.93, p<0.0001), all factors were significant (Table 2). With steeper slopes, the amount of former forest occupied by salt marsh was higher, although these areas experienced the lowest rates of forest loss overall (Figure 2). For the local salinity gradient, areas closer to the local head of tide exhibited lower rates of salt marsh migration. Similarly, for the estuary-wide salinity gradient, areas further up the bay toward the Delaware River exhibited less marsh migration (Figure 3). The interaction between local and regional salinity indicated that the local gradient became less influential further up the bay as overall salinity decreased (Figure 4).

**Table 2:** Results of Generalized Linear Model for the effect of slope and salinity on the amount of marsh migration between 1930 and 2006.

Independent variable	Estimate	Std. Error	*t-Value*	*p-Value*
Intercept	−0.2409	0.1366	−1.7635	0.0863
Estuary-wide salinity gradient	0.0033	0.0011	2.9567	0.0055
Local salinity gradient	0.0754	0.0255	2.9581	0.0054
Local x estuary-wide salinity gradient	0.0043	0.0011	4.0225	0.0003
Slope	0.2165	0.0692	3.1290	0.0035

## Discussion

Over a 76-year time frame, I found that at the salt marsh-forest interface, forest has been lost at more than three times the amount (1.8 m/yr) that salt marsh has been gained via inland migration (0.54 m/yr). On the the adjacent Delaware Bay coastline at this study site, loss of salt marsh to erosion is approximately 3 meters per year [37], an amount that is 5.5 times greater than the amount being gained by inland migration (0.54 m/yr). This shoreline erosion rate surpasses that of Louisiana (∼0.8–1.38 m/yr) [38], [39], an area that is experiencing some of the highest rates of salt marsh erosion in North America [40], [41] and is equivalent to the rate of erosion documented there during the peak of BP–*Deepwater Horizon* oil spill impacts (∼3.0 m/yr) [39]. The only habitat in this system that is experiencing a net increase is *Phragmites*-dominated wetland.

The lag in habitat change at the salt marsh-forest interface is due to the mediating effect of *Phragmites*, which quickly invades disturbed areas created by dying trees at the salt marsh ecotone [23], [24] via clonal expansion from the salt marsh edge [22] becoming the dominant plant in these areas. In order for forest to become salt marsh, forest must first die back and be invaded by *Phragmites*. The *Phragmites* must then yield to salt marsh as soil conditions become more saline [42], [43].

The difference in the pace of change between forest and *Phragmites*-dominated wetland is likely due to their differing capacities to resist change. *Phragmites* can resist change longer because it has a broad range of tolerance to varying salinity and moisture regimes [42], [43], it has the ability to accrete [44], [45] along with the ability to seek less saline water via clonal expansion inland [22]. Forests have a much narrower range of tolerance to moisture and particularly salinity [46], and thus succumb to change at a faster rate than *Phragmites*. Only when salinity reaches an upper threshold does *Phragmites* yield to the inland encroachment of salt marsh.

This study's findings suggest that the capacity of *Phragmites* to slow the progress of inland salt marsh migration is influenced by geographic patterns of salinity variation. Areas with lower salinity experienced the least salt marsh migration, whereas higher salinity areas experienced higher rates of salt marsh migration. These higher salinity areas are in the lower bay (salinity approximately 25–30), while lower salinity areas are found in the upper reaches of small coastal streams and in the middle and upper estuary (salinity approximately 17–25). No *Phragmites* expansion into existing salt marsh was evident in the study area during the time frame of the study.

The significant effect of slope in the analysis of salt marsh migration (Table 2) appears at first to be counterintuitive (i.e. that there is greater salt marsh migration at steeper slopes). A likely reason that a larger proportion of the former 1930 forest was occupied by salt marsh in these steeper areas is that *Phragmites* was “squeezed” [47] between steeper uplands and saline wetlands. This suggests that, in these steeper areas, *Phragmites* is no longer progressing inland and that forest dieback may be progressing slowly in these areas.

Variation in forest dieback was predicted by local slope, with lower slopes experiencing the greatest amount of forest loss. The small sample of low-slope coastal peat basins dominated by Atlantic white cedar (n = 4) experienced some of the greatest amount of forest loss in the study area. The dramatic increase in forest edge over the 76-year timeframe of the study (due to increasing dissection of the edge) indicates that increasing numbers of trees are exposed to the zone of active change at the forest-salt marsh ecotone. This observation suggests that a positive feedback mechanism may exist that could accelerate the rate of change as forest edge increases.

Seasonal and annual fluctuations in precipitation, storm frequency, and changes to groundwater hydrology may influence temporal variation in habitat change rates [13]. While I depict here a record of change between two points in time, the habitat changes documented may have occurred both gradually, and in pulsed events, particularly for the more sensitive forest habitats. The role of storms and drought in forcing change needs further study.

Sea level rise is hypothesized to be the ultimate driver of these changes and the steady increase of relative sea level rise in the region is well-documented [14]. Nonetheless other factors may play a role in the forest dieback documented here. In particular, changes to hydrology in coastal wetlands via mosquito ditching may bring greater amount of tidal flux to upper reaches of marshes [48] and reduced freshwater discharge due to groundwater withdrawal may also promote intrusion of salt water into upland locations [49]. Regardless of the cause of increasing salinity and/or moisture at the salt marsh ecotone, the resulting habitat changes are likely to be similar.

### Implications for climate adaptation

The east coast of North America will be experiencing among the most rapid rates of sea level rise in the world [14], [50]. To conserve salt marshes to the maximum extent possible, a variety of strategies must be employed [51]. One of the most widely embraced strategies [52]–[55] is to maintain coastal uplands in natural cover to allow for transgression of tidal wetlands into uplands as sea level rise increases. This study's findings that sea level rise differentially favors the expansion of an invasive species as transgression into uplands proceeds has important implications for the future because some of the functions of tidal marshes may be lost (e.g. habitat for salt marsh dependent species [56], [57]) while others may be maintained (e.g. storm buffer for uplands [45]). Invasive plants are affecting estuarine ecosystems throughout the world (e.g. *Spartina alterniflora* in China [58], mangroves in Hawaii [59]) and continued investigation may reveal that sea level rise is influencing the distribution of other types of invasives, which may impact wetland function in these areas as well.

Understanding the effect of rising sea levels on the distribution and abundance of native and invasive species can be used to improve sea-level rise impact forecasting of habitat change for estuaries. Throughout the range of invasive *Phragmites* [60] variation in salt marsh migration due to salinity can be incorporated into models to yield more realistic predictions of habitat change. Improved predictions will help land use planners and managers target for protection those upland areas with the greatest likelihood of becoming salt marsh and will provide an improved understanding of how ecosystem function will be affected as proportions of invasive vs. native dominated wetlands change.

Results from this study indicate that protecting uplands to allow for salt marshes to move inland [61] may only fractionally offset the loss of existing marshes. In a recent 5 year period (2004–2009) 2.8% (45,140 hectares) of emergent tidal wetlands in the United States were lost [41]. This represents a three-fold increase in the rate of loss compared with the previous 5-year period [62]. Contrary to historic patterns of wetland change, the loss was not attributable to direct human impacts. Instead 99% of these losses were the result of the effects of storms and sea level rise [41]. The results of this study provide a clearer picture of how much salt marsh an upland protection strategy will help create and where these salt marshes are most likely to be created. While protecting uplands adjacent to these wetlands remains a key strategy for sea level rise adaptation planning, additional, complementary strategies [51], [63], [64] are needed to help mitigate continuing estuarine wetland losses.
